# Neuroglobin Expression Models as a Tool to Study Its Function

**DOI:** 10.1155/2019/5728129

**Published:** 2019-06-19

**Authors:** Evi Luyckx, Zoë P. Van Acker, Peter Ponsaerts, Sylvia Dewilde

**Affiliations:** ^1^Protein Chemistry, Proteomics and Epigenetic Signalling, University of Antwerp, Antwerp B-2610, Belgium; ^2^Laboratory of Experimental Hematology, Vaxinfectio, University of Antwerp, Antwerp B-2610, Belgium

## Abstract

Neuroglobin (Ngb) is an evolutionary conserved member of the globin family with a primary expression in neurons of which the exact functions remain elusive. A plethora of *in vivo* and *in vitro* model systems has been generated to this day to determine the functional biological roles of Ngb. Here, we provide a comprehensive overview and discussion of the different Ngb models, covering animal and cellular models of both overexpression and knockout strategies. Intriguingly, an in-depth literature search of available Ngb expression models revealed crucial discrepancies in the outcomes observed in different models. Not only does the level of Ngb expression—either physiologically, overexpressed, or downregulated—alter its functional properties, the experimental setup, being *in vitro* or *in vivo*, does impact the functional outcome as well and, hence, whether or not a physiological and/or therapeutic role is ascribed to Ngb. These differences could highlight either technical or biological adaptations and should be considered until elucidation of the Ngb biology.

## 1. Introduction

Gaining insight into the homeostatic mechanisms in the brain that support the maintenance of pivotal cell survival-promoting mechanisms has been very valuable for the development of therapeutic strategies for the treatment of nervous system-related injuries and diseases. An essential factor in the cellular metabolism of this highly metabolically active tissue is oxygen (O_2_), a key player in cell growth and survival. Both the up- and downregulation of O_2_ tension has a pronounced effect on brain function [1]. While the former is responsible for the production of reactive oxygen species (ROS), which are key players in oxidative stress metabolism, reduced O_2_ tension can also be life-threatening. Depending on the O_2_ tension, the brain adapts its approach to endogenous protective mechanisms and neuroplasticity to support cell survival and homeostasis.

The tight regulation of O_2_ tension and levels of harmful O_2_ derivatives is partially controlled by a phylogenetically widespread family of haem-containing proteins called “globins”. The presence of the haem allows globins to bind diatomic gases (O_2_, CO, and NO), which enables them to perform a variety of functions, including O_2_ sensing, transport, and storage, haem-based catalysis, and the scavenging of reactive oxygen and nitrogen species (ROS/RNS) [2, 3]. Globins associated with or expressed in nervous tissue are referred to as “nerve globins” and have been reported in both vertebrates and invertebrates [4–7]. In 1872, Lankester was the first to report a nerve globin in the nerve cord of the polychaetes annelid *Aphrodite aculeate* [6]. Cytoglobin, haemoglobin (Hb), and myoglobin (Mb) had been reported to be coexpressed in the mammalian nervous system in addition to their primary tissue-specific expression patterns. However, in 2000, Burmester and colleagues were the first to discover a globin type that is predominantly expressed in human and mouse brains: neuroglobin (Ngb) [4, 8–12].

## 2. Ngb: A Conserved Cytoprotective Protein

### 2.1. Structure and Reactivity of Ngb

Ngb is an aberrant member of the globin family, featuring only 20 to 25% protein sequence identity to Hb and Mb [4]. Despite its sequence differences, Ngb has a monomeric structure and exhibits the classical three-over-three *α*-helical globin fold that forms a hydrophobic pocket around the haem [13]. Given the high sequence identity (94%) between human Ngb [13] and mouse Ngb [14], it may be of no surprise that they are structurally alike. In addition, with the high 1.5 Å resolution obtained with mouse ferric Ngb crystals, Ngb could be modelled as a molecular fossil with various binding and entry options to the central haem iron [14]. Intriguingly, in contrast to the classical pentacoordinated globins, the Fe^2+^ deoxy and Fe^3+^ state of the Fe atom of Ngb's haem are able to be hexacoordinated. In the absence of an external ligand, the sixth distal position of the haem Fe is bound to the histidine at position 7 of the E-helix (HisE7). Due to hexacoordination of the haem Fe, external ligands (O_2_, CO, and NO) are subjected to an intrinsic binding competition with HisE7, of which the functional significance is not yet fully understood [13, 15–17].

Hb and Mb are structurally constructed to support O_2_ transport and storage, whereas Ngb is characterised by only a moderate O_2_-binding affinity under physiological conditions (*P*_50_ = 7.5 Torr at 37°C and neutral pH) [18]. Given their high metabolic rate, neurons are required to experience cellular *P*_50_ levels close to zero which means that, under *in vivo* conditions, the fractional O_2_ saturation of Ngb in vertebrates would be too low to play a role in O_2_ transport or storage [18]. Furthermore, the relatively low Ngb concentration in the brain (±1 *μ*M) provides only limited Ngb capacity to bind, transport, or store O_2_ molecules [4]. Therefore, the physiological functions of Ngb might not be primarily related to oxygen level preservation but rather to the scavenging of noxious ROS and RNS, which accumulate in the cell after hypoxic or ischemic insults and subsequent reperfusion of the tissue [19].

Ngb's sequence has been well-conserved during mammalian evolution. It has a threefold slower evolutionary rate compared to Hb and Mb, and its resemblance to some invertebrate nerve globins suggests a link to a very old globin lineage. Hence, these data support a strongly selected, important functional role of Ngb [4, 20].

### 2.2. Ngb's Expression Patterns and Mode of Neuroprotection

As its name suggests, Ngb is predominantly expressed in neurons of the central and peripheral nervous systems. Recent reports have shown that the highest Ngb levels are found in the hypothalamus, confirmed at both the transcript and protein levels, where *Ngb* mRNA expression is even up to 100-fold higher compared to the cerebral cortex, cerebellum, and hippocampus, which were initially considered to be primary Ngb expression sites. This regional peak of Ngb expression has also been shown to be conserved in humans and other mammals [21, 22]. Additionally, high Ngb concentrations have also been reported in other nonneural high metabolically active or specialised tissues, including the retina and several endocrine tissues such as the adrenal and pituitary glands. However, *Ngb* mRNA expression levels in the retina and testes are low, according to a study by Fabrizius et al. [4, 22–24]. An important concern in the debate of Ngb expression sites is the potential discrepancy between Ngb mRNA and protein expression levels that has to be considered. Furthermore, Ngb has been found to undergo several transcriptional and translational modifications, such as epigenetic and posttranslational modifications which may regulate final Ngb expression levels [25, 26]. Subcellular cytoplasmic localisation of Ngb was undisputed for a long time, as Ngb mRNA and protein signals were consistently detected in perikarya, axonal processes, axonal varicosities, and terminal synapses [23, 27–30]. However, recently, Ngb has also been reported to be expressed in the nuclei of neurons and in the inner wall of mitochondria under certain cellular conditions [31, 32].

**Figure 1:**
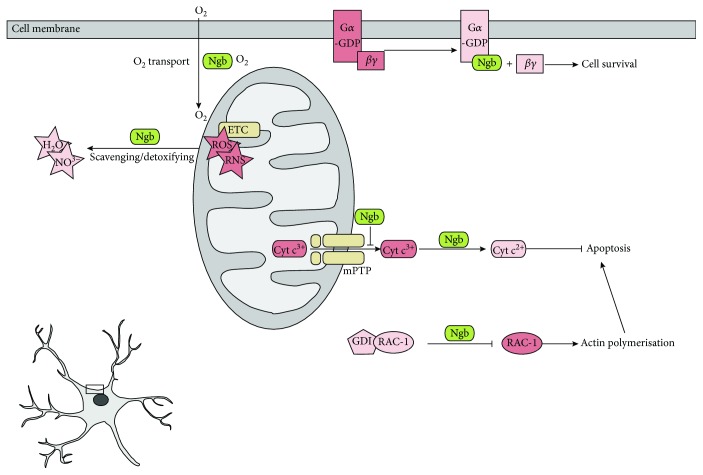
Overview of the potential Ngb neuroprotective mechanisms in neurons. The schematic presentation is a magnification of the boxed area in a neuron. Ngb is detected to detoxify harmful excesses of NO and to scavenge ROS and RNS amongst others by its association witch Cyt c_1_. Mitochondrial translocation suggested by the latter is exerted by HTT. Ngb has a guanosine nucleotide dissociation inhibitor (GDI) activity and can prevent G*α* from binding to the G*βγ* complex, which promotes neuronal survival. Ngb also may inhibit the dissociation of RAC-1 from its endogenous GDI, preventing actin polymerisation and microdomain aggregation. Furthermore, Ngb might inhibit opening of mPTP pores of mitochondria and subsequent Cyt c (Fe^3+^) release. Ngb also converts apoptotic Cyt c (Fe^3+^) to Cyt c (Fe^2+^). Furthermore, Ngb might modulate the AKT/IP3 signalling pathway and associate with Na^+^/K^+^ ATPase in order to promote neuroprotection. Lastly, while Ngb is able to bind oxygen, its oxygen affinity value only attains 7.5 Torr. Given this value to be lower than the O_2_ tension within neurons, it is unlikely that Ngb exerts a respiratory function under basal conditions. Potentially harmful species are indicated in dark pink and converted nonharmful species in light pink. Ngb: neuroglobin; ROS: reactive oxygen species; RNS: reactive nitrogen species; ETC: electron transport chain; GDI: guanosine nucleotide dissociation inhibitor; Cyt c: cytochrome c; mPTP: mitochondrial permeability transition pore; GDP: guanosine diphosphate; RAC-1: ras-related C3 botulinum toxin substrate 1; Cyt c_1_: cytochrome c_1_; HTT: huntingtin; VDAC: voltage-dependent anion channel 1; IP3: inositol triphosphate; PIP3: phosphatidylinositol (3,4,5)-trisphosphate.

As Ngb expression sites may vary, recent studies point to distinct Ngb functions for high- and low-expressing cells and tissues [22]. To date, Ngb is considered to exert neuroprotective actions through different molecular mechanisms, of which the major aim is to promote cellular homeostasis and survival [4, 15, 33, 34] (Figure 1). It is unlikely that Ngb acts as a classical globin to enhance O_2_ supply to the mitochondria of metabolically active neurons to enhance the respiratory system. Although Ngb is predominantly expressed in metabolically active cells, cellular Ngb concentrations and Ngb oxygen affinity are too low, thus being unsuitable to support this hypothesis as previously described [4, 18]. Secondly, Ngb is believed to act as a detoxifier of harmful excesses of NO and as a scavenger of ROS and RNS, thereby reducing oxidative stress [19, 35] (Figure 1). The interaction of Ngb with cytochrome c_1_ (Cyt c_1_), a subunit of the mitochondrial complex III, might be of great importance in the latter, as complex III is a component of the respiratory chain and a major source of ROS and RNS [36]. Furthermore, Ngb might affect the intrinsic apoptotic pathway at several stages (Figure 1). Ngb has been reported to inhibit the opening of mitochondrial permeability transition pores (mPTP) by binding to one of its key components, namely, the voltage-dependent anion channel 1 (VDAC), and inhibiting subsequent proapoptotic cytochrome c (Cyt c) (Fe^3+^) release [36, 37]. In addition, by sequestering Cyt c (Fe^3+^) and forming a Ngb (Fe^2+^)-Cyt c (Fe^3+^) complex, Ngb (Fe^2+^) reduces Cyt c (Fe^3+^) to Cyt c (Fe^2+^) through a redox reaction and decreases the release of proapoptotic Cyt c (Fe^3+^), thereby supporting cell survival [38]. This process requires high levels of Ngb and its translocation close to the mitochondria [32, 39, 40]. An important mediator that was reported for Ngb trafficking from the cytosol to the mitochondria is the association of Ngb with huntingtin (HTT) [41, 42]. Thus, the involvement of Ngb in respiratory chain functions and the regulation of the intrinsic apoptosis pathway may be important for its neuroprotective function. It is to be noted that for Ngb to function as a radical scavenger or cytochrome c reductase, the cell is to provide in an electron donor for Ngb that can reduce it again into its ferrous form (Fe^2+^). However, such an electron donor is yet to be discovered [34]. Hence, the exact mechanisms or implications of Ngb's interaction with Cyt c remain elusive.

In addition, several interactions of Ngb with signalling proteins have been described, suggesting a potential regulatory role of Ngb in the modulation of cell signalling [43]. Firstly, Ngb might act as a heterotrimeric G*α* protein guanosine nucleotide dissociation inhibitor (GDI). The interaction of Ngb (Fe^3+^) with guanosine diphosphate (GDP)-bound G*α* could then protect against neuronal death, as the interaction of Ngb (Fe^3+^) with the GDP-bound G*α* subunit inhibits G*α* activity and prevents G*βγ* rebinding to G*α*, thereby enhancing the survival-promoting G*βγ*-dependent pathway that acts through activation of phosphatidylinositol 3-kinase (PI3K) [44, 45] (Figure 1). Furthermore, it has been reported that the activity of Ngb is not limited to interactions with heterotrimeric G proteins. Ngb might also be important for the regulation of small GTP-binding proteins of the Rho family. It has been reported that Ngb inhibits PAK1 kinase and interacts with members of the RhoGTPase family and with the Rho GDP dissociation inhibitor (Figure 1). As such, Ngb inhibits hypoxia or N-methyl-D-aspartate- (NMDA-) induced death signals that trigger reorganisation of the cytoskeleton and polarisation of lipid raft membrane microdomains, as well as associated mitochondrial aggregation [46, 47]. The interest in the mitochondrial localisation of Ngb has grown in recent years as it may play a role in the neuroprotective action of Ngb [36, 48, 49]. Reallocation of Ngb to the mitochondria and its subsequent neuroprotective effects have also been observed after treatment with 1-methyl-4-phenylpyridinium ion in Ngb-overexpressing SK-N-BE2 cells, where its neuroprotective effect was linked to mitochondrial lipid raft-associated complexes [50]. However, the exact mechanism regulating this reallocation remains unclear, as Ngb lacks a mitochondrial signalling sequence. Another important modulation of intracellular signalling by Ngb is the activation of the serine/threonine kinase (AKT) pathway, which has been reported to have several neuroprotective actions in several life-threatening insults [51–54]. In addition, interactions of Ngb with AKT and its upstream regulator PTEN have been reported in neuritogenesis, suggesting a role for Ngb as an upstream regulator of the PI3K/AKT pathway [55]. Furthermore, the recently described interaction of Ngb with the Na^+^/K^+^ ATPase *β*1 subunit also holds great promise, as Ngb preserves its activity [56]. Other Ngb-associated prosurvival mechanisms are still being elucidated to explain Ngb's neuroprotective function by modulating different pivotal cellular processes [57, 58].

Since its discovery, research groups have been investigating the structure, reactivity, expression patterns, localisation, and functional significance of Ngb. Despite the fact that more than 500 studies have been published on Ngb, its exact mechanisms of action that underlie its neuroprotective actions remain to be elucidated.

## 3. Animal Models of Ngb Modulation

**Figure 2:**
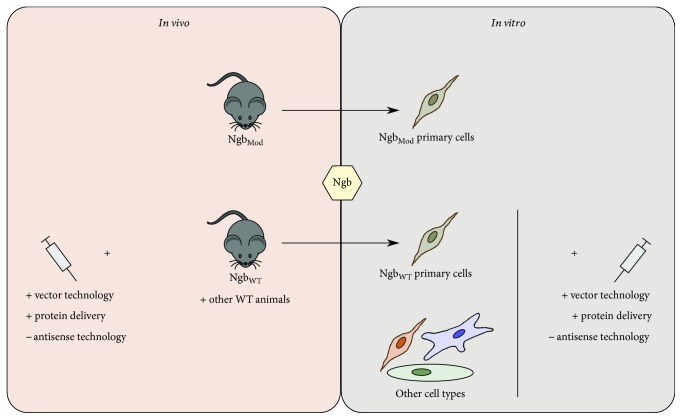
Overview of the different types of Ngb expression models. The *in vivo* models comprise animal models of modulated Ngb expression (Ngb_Mod_) on the one hand (i.e., Ngb-overexpressing and Ngb-deficient mouse models) and WT animals on the other hand. Ngb overexpression in WT animals can be obtained by injection of Ngb-expressing vectors or protein delivery. Ngb knockdown can be established by injection of *anti-Ngb* RNAs. The *in vitro* models comprise primary cells derived of the Ngb-modulated mouse models, primary cells from WT animals, and other cell types. To create Ngb overexpression or Ngb deficiency, similar techniques can be used as with the *in vivo* models.

As endogenous Ngb levels are low, many *in vitro* and *in vivo* Ngb expression models have been created to provoke essential stimuli to investigate Ngb's response mechanisms upon different life-threatening insults. The development of Ngb-overexpressing or Ngb-deficient models (Figure 2) has already enabled researchers to gain more insight into the significance of Ngb in wild type (WT) *in vivo* and *in vitro* systems. In addition, this has also led to a better understanding of the neuroprotective role of Ngb in several central nervous system pathologies, such as Alzheimer's disease, Parkinson's disease, and Huntington's disease, brain ischemia and hypoxia, neurodegeneration, traumatic brain injury, and cancer models [34].

### 3.1. Transgenic Ngb Overexpression Models

**Figure 3:**
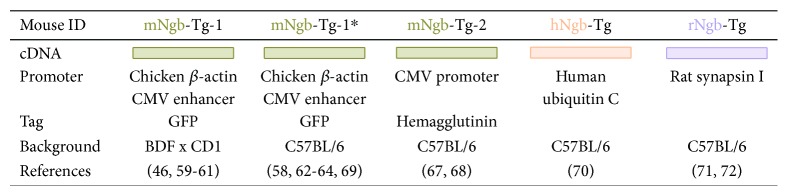
Overview of transgenic Ngb-overexpressing mouse models. CMV: cytomegalovirus; GFP: green fluorescent protein. ^∗^Indicating that this line was made congenic. Murine Ngb models are presented in green, human Ngb models in orange, and rat Ngb models in purple.

**Figure 4:**
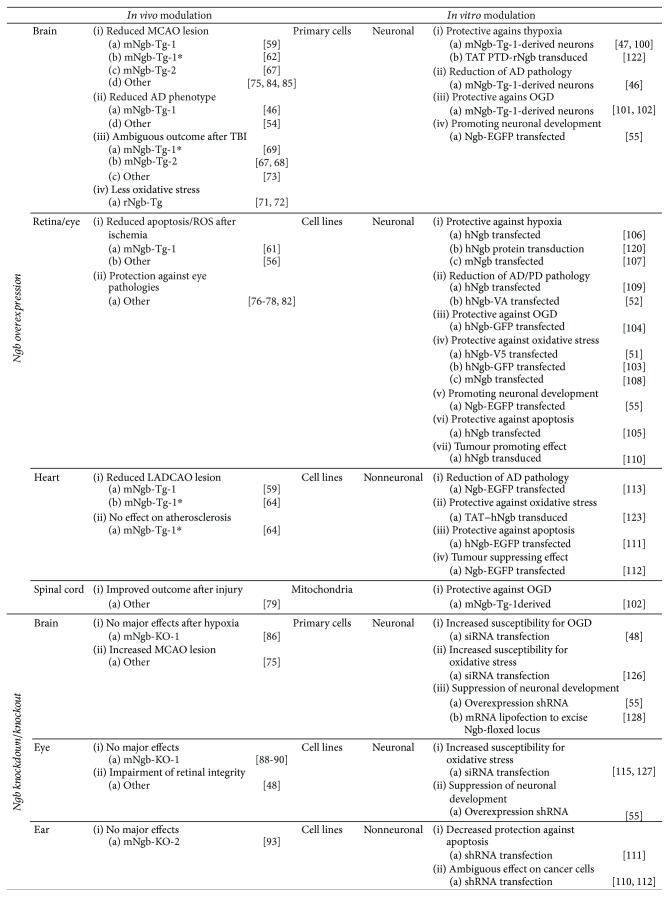
Overview of in vivo and in vitro modulation of Ngb expression and their tissue-specific outcome.

Approximately ten years ago, Khan and colleagues were the first to report the development of a Ngb-overexpressing transgenic mouse line, referred to as mNgb-Tg-1 (Figure 3). Full-length murine Ngb cDNA was cloned in a pTR-UF12d vector downstream of the chicken *β*-actin promoter and cytomegalovirus (CMV) enhancer and upstream of Renilla reniformis green fluorescent protein (GFP). The final construct was digested and microinjected into fertilised eggs of BDF x CD1 mice. As the chicken *β*-actin promoter is tissue nonspecific, enhanced Ngb expression in homozygotes is widespread. It covers multiple cell types and tissues, including the heart and brain-specific cells such as neurons, astrocytes, and endothelial cells in the cerebral cortex [59, 60]. Furthermore, mNgb-Tg-1 mice are viable and do not show any physical or behavioural abnormalities. As western blot analyses demonstrated increased Ngb protein levels in the heart and brain of homozygotes, mNgb-Tg-1 mice are frequently used in studies of cerebral and myocardial ischemia, stroke, and neurodegenerative diseases [46, 59, 60]. Ischemic studies on mNgb-Tg-1 mice revealed that cerebral infarct volumes after occlusion of the middle cerebral artery (MCAO) were reduced by 30%, compared to the WT. The volume of myocardial infarcts, produced by occlusion of the left anterior descending coronary artery (LADCAO), was reduced by 25% ([59], Figure 4). Furthermore, studies on retinal ischemia revealed that Ngb overexpression was beneficial against retinal ischemia-reperfusion injury, by decreasing mitochondrial oxidative stress-mediated apoptosis. This effect is likely due to an 11.3-fold higher Ngb mRNA expression in mNgb-Tg-1 mice than in WT controls ([61], Figure 4). Ngb expression was found to be localised within the mitochondria of the ganglion cells, outer and inner plexiform layers, and photoreceptor inner segments, which supports the evidence of widespread Ngb expression caused by the chicken *β*-actin promoter [61]. Alzheimer's disease research on mNgb-Tg-1 x APP (Sw, Ind) (amyloid precursor protein) double transgenic mice showed that increased Ngb levels reduce amyloid beta (A*β*) deposits, decrease levels of A*β*(1-40) and A*β*(1-42), and improve behavioural performance, thereby abating the Alzheimer's disease phenotype ([46], Figure 4).

Subsequently, The Jackson Laboratory backcrossed these mice to a C57BL/6J background for at least five generations to generate the congenic commercially available strain: B6.Cg-Tg(CAG-Ngb,-EGFP)1Dgrn/J (007575, The Jackson Laboratory) or mNgb-Tg-1∗ ([62], Figure 3). By analogy with the original mNgb-Tg-1 mouse model, the derived congenic strain was used in different ischemic and hypoxic setups [62–64]. Although Ngb's cytoprotective function was confirmed during acute myocardial infarction, this effect was less pronounced in a mNgb-Tg-1∗ atherosclerosis model, where Ngb overexpression did not affect survival nor occurrence of myocardial infarcts ([64], Figure 4). Intriguingly, for cerebral ischemia, a significant reduction in brain infarct volume was observed 24 hours after ischemia in mNgb-Tg-1∗ mice, but the infarct volume was found to be specific to the genetic background of the mice [62]. Thus, care must be taken when comparing different studies using mNgb-Tg-1 mice of either the original or congenic line, as different experimental outcomes could be achieved. Furthermore, caution must be taken when crossbreeding these specific mouse lines with transgenic Alzheimer mice or atherosclerotic mice, as it is known that one transgene may produce a severe phenotype in one strain and a milder one in another strain. Therefore, moving alleles from one background (BDF x CD1) to another (C57BL/6) or combining different transgenes could complicate the interpretation and comparison of studies. Hence, we suggest that researchers pay close attention to the mouse model's genetic background, as is already routinely done for their homozygous and heterozygous states [62, 65, 66].

Wang and colleagues have also produced a transgenic mouse line, referred to as mNgb-Tg-2, which overexpresses murine Ngb fused to the N-terminal hemagglutinin epitope tag under the control of a CMV promoter. It was initially created in a B6C3F1 background and subsequently crossed with C57BL/6 mice ([67], Figure 3). Analogous to the chicken *β*-actin promoter, the nontissue-specific CMV promoter in mNgb-Tg-2 mice provides enhanced Ngb expression in neurons and other cell types, such as astrocytes [67]. Although transient focal cerebral ischemia led to reduced brain infarction volumes in mNgb-Tg-2 mice, as expected from similar results in mNgb-Tg-1 mice, the response for traumatic brain injury (TBI) was different ([67, 68], Figure 4). While Ngb overexpression in mNgb-Tg-1 mice improved sensorimotor outcomes, the recovery of sensorimotor and spatial memory functional deficits was not improved in mNgb-Tg-2 mice. Nonetheless, traumatic lesion volume was also reduced in mNgb-Tg-2 mice [68, 69]. This observation suggests that despite using tissue nonspecific promoters, studies on mNgb-Tg-1 versus mNgb-Tg-2 mice might have a different outcome based on the difference in transgenic construct. Furthermore, Wang et al. observed Ngb protein levels to be 1.5-fold higher in mNgb-Tg-2 mice as compared to WT controls. Of note, the mNgb-Tg-2 Ngb level was relatively lower than when the mouse line was first generated five years ago [67]. This decline in Ngb expression levels may be caused by endogenous depletion of inserted exogenous DNA fragments or by inactivation of the CMV promoter [68]. Therefore, it is crucial to carefully assess Ngb protein levels in transgenic models on a regular basis to assure the reproducibility of the model. Furthermore, Zhao et al. used a 10-point neurological severity score to assess post-TBI neurological dysfunction, while Taylor et al. used the grid walk test. The neurological severity score evaluates the ability to walk rather than the accuracy of locomotion. As it does not take foot faults into account, this test is less sensitive and might discount this important impairment [69]. In addition, both studies used different controlled cortical impact injury sites and impact parameters, which might influence the severity of the TBI lesion.

Li and colleagues produced a third *in vivo* Ngb-overexpression mouse model. Their hNgb-Tg mouse line overexpresses human Ngb under the control of the human ubiquitin C promoter, expressing Ngb ubiquitously in neural and nonneural tissues such as the brain, heart, and kidneys ([70], Figure 3). Ngb's protein levels are 3-fold higher than that in WT mice, providing neuroprotection. It was confirmed in this mouse model that CA1 neuronal injury after hippocampal ischemia-reperfusion was significantly reduced as compared to WT mice, and there was a decrease in CA1 hippocampal ROS/RNS production and lipid peroxidation ([70], Figure 4). These results are in line with the previously discussed results of the mNgb-Tg mice.

The final reported Ngb overexpression mouse model, called rNgb-Tg, was produced by Lee and colleagues and expresses rat Ngb under the control of a neuronal-specific rat synapsin I promoter ([71], Figure 3). To date, this mouse model has only been used to assess the neuroprotective effects of Ngb on the mouse brain in a model of acute inhalation of combustion smoke, which generates oxidative stress in the brain. The rNgb-Tg mouse model revealed that Ngb overexpression alleviates mitochondrial impairments and oxidative DNA damage formation that is caused by combustion smoke inhalation ([71, 72], Figure 4).

### 3.2. *In Vivo* Ngb Overexpression and Delivery

In order to introduce ectopic expression of proteins in various cell types and tissues, viral gene delivery systems based on adenoviruses, adenoassociated viruses, and lentiviruses have been of great value (Figure 2). To determine the functional significance of Ngb, different systems have been used over the past years. An adenoviral construct, pAd-GFP-rNgb, was used to investigate the neuroprotective effect of rNgb overexpression on TBI in rats [73]. By using this adenoviral vector 5, genes can be transferred to both dividing and nondividing cells with a broad range of infectivity, low host specificity, and high tissue transgene levels. Furthermore, this vector remains epichromosomal and thus does not integrate into the host genome. However, its immunogenic character has limited its use in clinical applications [74]. To overcome this immunogenic effect, adenoassociated vectors could be used, providing a high and long-term expression level *in vivo* and evoking a very low immune response. Adenoassociated vectors resemble adenoviral vectors in their remaining features but provide a limited transgene capacity. Particles can contain up to 4.8 kb compared to 7.5 kb in adenoviral vectors [74]. Sun et al. intracerebrally injected a pTR-UF12d-mNgb-GFP vector with a CMV enhancer and *β*-actin promoter and with GFP as a reporter gene into the cerebral cortex and striatum of mice, successfully resulting in increased expression of Ngb in cerebral cortical neurons. This Ngb overexpression reduced infarct size and improved functional outcomes after an ischemic insult caused by MCAO ([75], Figure 4). Additionally, different studies on eye pathologies applied variations on an adenoassociated-2/2-mNgb vector to administer Ngb overexpression by subretinal or intravitreous injection. Ocular Ngb levels were shown to be critical in retinal homeostasis and cellular preservation ([76–78], Figure 4). In a rabbit model of spinal cord injury, lentivirus-mediated Ngb overexpression was successfully obtained by injection of Lv-rbNgb-EGFP into the spinal cord. Ngb overexpression mediated improvements in spinal cord injury outcomes and reduced secondary damage ([79], Figure 4). Furthermore, Wen *et al.* showed that a Ngb lentiviral vector effectively ameliorated postischemic neuronal death in CA1 in the rat hippocampus [56]. Lentiviral vectors are an interesting vehicle for gene transfer due to their ability to integrate into the genome of nondividing or dividing host cells and to deliver up to 8 kb of content. Due to their low immunogenicity, high-efficiency infection, long-term stable expression, and neural stem cell- (NSC-) infecting preference, they are a valuable tool in neurorelated research [79]. Another alternative method for achieving Ngb overexpression with vector technology was described by Li and colleagues, who used an intracerebroventricular injection of a pcDNA3.1-mNgb construct in APP/PS1 (presenilin-1) transgenic Alzheimer mice. Although A*β* deposition and production were attenuated after injection of this simple mammalian expression vector, no data were reported about the efficiency of transfection and level of Ngb overexpression. Therefore, these data should be interpreted with caution ([54], Figure 4).

Alternatively, Ngb delivery can be obtained by engineering Ngb at the protein level. Sugitani et al. designed a recombinant chimeric Ngb consisting of four modules encoded by four exons. The first exon of human Ngb (**H**HHH, H = human module) was replaced with zebrafish Ngb (**Z**ZZZ, Z = zebrafish module) as the latter is known to be a cell membrane-penetrating module, creating the chimeric **Z**HHH Ngb [80, 81]. Intraocular injection of this recombinant cell membrane-penetrating human Ngb protein into the mouse eye led to a 2-fold increase in Ngb expression in retinal ganglion cells, promoting retinal ganglion cell survival and optic nerve regeneration after optic nerve injury ([82], Figure 4). Another technology to cross membranes, including the blood-brain barrier, consists of using cell-penetrating peptide (CPP) delivery. Fusion proteins with the 11-amino-acid human immunodeficiency virus transactivator of transcription (TAT) protein transduction domain showed successful delivery of macromolecules into the brain [83]. Systemic injection of TAT-mNgb successfully resulted in increased Ngb levels in neurons of the mouse brain and increased neuronal survival after MCAO ([84, 85], Figure 4).

### 3.3. Ngb-Deficient Models

**Figure 5:**
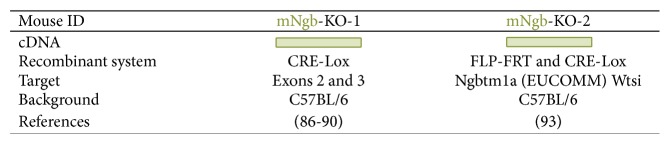
Overview of Ngb-deficient mouse models. Mouse Ngb models are presented in green.

Hundahl and colleagues were the first to describe a Ngb knockout mouse model, mNgb-KO-1. It was generated by crossbreeding a Ngb-floxed (Ngb_fl_) mouse, in which *loxP* sites were introduced into the introns flanking exons 2 and 3 of the *Ngb* locus, with a mouse model expressing CRE recombinase under the CMV promoter ([86], Figure 5). Loss of exons 2 and 3 of the *Ngb* locus prevents Ngb expression [86]. With this mouse model, Hundahl et al. reported that Ngb deficiency provokes *Hif1A* and *c-Fos* responses and thus lowers the threshold for hypoxia-induced gene expression. However, it had no effect on neuronal survival following acute and prolonged hypoxia in mNgb-KO-1 mice ([86], Figure 4). Intriguingly, although Ngb overexpression studies mainly describe the neuroprotective effect of Ngb, ambiguous results have been reported in Ngb-deficient models, suggesting that Ngb expressed at endogenous levels does not have a neuroprotective function in ischemia *in vivo* [86, 87]. Furthermore, the functional significance of endogenous Ngb in the retina remains unclear. Endogenous Ngb is not thought to play a major role in retinal oxygen homeostasis and only has a minor effect on light-dependent gene expression ([88], Figure 4). These observations suggest only a subtle systemic role for Ngb. In addition, Ngb is not thought to affect general circadian behaviour but it evokes an increased behavioural response to light in the suprachiasmatic nucleus, in conjunction with increased *Per1* gene expression ([88–90], Figure 4).

As several studies already suggested the presence of Ngb in the peripheral and central structures of the auditory systems of rats, mice, and humans, Nowotny and colleagues recently explored a new Ngb knockout model to determine the role of Ngb in the auditory system [91–93] (Figure 5). This mNgb-KO-2 mouse model was designed by using C57BL/6N-derived embryonic stem cell technology, comprising of a promoter-driven gene targeting cassette: Ngbtm1a(EUCOMM)Wtsi. Crossbreeding of mice carrying this gene targeting cassette with FLP- and CRE-deleter strains resulted in a Ngb knockout mouse referred to as mNgb-KO-2 [93, 94]. The lack of Ngb in this mouse model resulted only in small deficits in hearing ability ([93], Figure 4).

### 3.4. *In Vivo* Ngb Silencing through Antisense Technology

To knock down gene expression, synthetic nucleic acids have been widely used in *in vitro* and *in vivo* setups over the past decades (Figure 2). The most common antisense gene silencing strategies are based on single-stranded antisense oligonucleotides or RNA interference (RNAi) and have a common aim: hybridisation with a unique target RNA sequence to block translation. In 2003, Sun et al. described the use of a phosphorothioate antisense oligodeoxynucleotide (PS-ODN), labelled with fluorescein isothiocyanate (FITC) at the 5′ end and directed against a part of the initial coding region of *mNgb* [75]. Intracerebroventricular injection of this *anti-mNgb* PS-ODN increased the infarct volume and aggravated the functional neurological outcome after focal cerebral ischemia induced by MCAO, which is in contrast with the results in Ngb overexpression models ([75], Figure 4). Furthermore, intraventricular administration of these PS-ODN sequences in a study by Wen et al. in 2018 showed that the neuronal damage after transient global cerebral ischemia was markedly aggravated in the CA1 of hypoxic postconditioned rats [56]. PS-ODNs are the majorly studied ODNs because of their relative ease of synthesis and nuclease stability. To obtain the latter, PS-ODNs have an S atom replacing the nonbridging O_2_ atom of the sugar phosphate backbone. This chemical modification greatly improves stability towards nuclease digestion and improves binding to serum proteins *in vivo*. Hence, an increased half-life and greater delivery are created. Transfection efficiency and antisense activity remain moderate. High levels of PS-ODNs are needed due to inadequate affinity for the target sequence, leading to increased nonspecific hybridisations. Apart from its ability to activate RNase H for degradation of mRNA, the phosphorothioate backbone is also known to cause cytotoxicity due to its high affinity to several proteins on the cell surface or in serum. The presence of nonspecific effects resulting from intrinsic activities of the backbone may complicate the elucidation of the biological effects of silencing the *Ngb* gene [95–97].

In recent years, RNAi has become an important player in the sequence-specific degradation of host mRNA. This technology is based on cytoplasmic delivery of dsRNA, such as short hairpin RNAs (shRNAs) identical to the target sequence, which can be degraded through an enzymatic pathway involving the endogenous RNA-induced silencing complex [98]. Transfection of shRNAs can be lipid-based through a plasmid vector encoding shRNAs transcribed by an RNA polymerase III or modified polymerase II promoter or through infection with virally produced vectors. In case of the latter, high and stable long-term expression can be obtained, as shRNAs are integrated into the host DNA. After transcription, shRNA is transported to the cytosol and interacts with the DICER enzyme to modify the molecule to be recognized by the RNA-induced silencing complex. Lechauve et al. injected *anti-Ngb* shRNA into the vitreous body of rats, which led to reduced activities of respiratory chain complexes I and III, degeneration of retinal ganglion cells, and impairment of visual function ([48], Figure 4). However, as the authors did not mention the exact shRNA system, these results should be evaluated with caution, keeping in mind that adenoviral delivery of shRNA can have toxic effects in mice [99]. For this reason, it is of great value that Lechauve et al. included a scrambled shRNA control in order to overcome ambiguous results [48].

## 4. *In Vitro* Models of Ngb Expression

### 4.1. Cell Lines Derived from Ngb Overexpression Mouse Models

A first method of obtaining Ngb-overexpressing cell lines is by culturing cells from Ngb genetically modified mouse models (Figure 2). Primary cortical neurons were prepared from 16-day-old mouse embryos of mNgb-Tg-1 and mNgb-Tg-2 mouse lines [46, 47, 100, 101]. This strategy allows the exploration of *in vivo* observations on a molecular basis *in vitro*. Khan et al. already reported the beneficial effects of Ngb overexpression in an *in vivo* model of Alzheimer's disease and subsequently demonstrated that Ngb overexpression showed resistance to the toxic effects of NMDA and A*β*(25-35) by preservation of several cellular processes ([46], Figure 4). Furthermore, they explored the regulation of the mechanism underlying the previously reported neuroprotective capacities of Ngb in hypoxic conditions ([47], Figure 4). Analogously, Wang et al. determined the effects of oxygen deprivation in the mNgb-Tg-2 mouse model and subsequently linked hypoxia-responsive genes to neuronal homeostasis and mitochondrial function ([100, 101], Figure 4). Another advantage of mouse model-derived cells consists of the nature of modification. Primary cortical neurons derived from transgenic mice were subjected to the transgenic modification during all the developmental stages while *in vitro* modifications only affect cells in the latest stadia, in an artificial manner.

### 4.2. Vector Technology in Ngb Overexpression Systems *In Vitro*

As many studies focus on the role of Ngb in neural-derived tissues *in vivo*, Ngb overexpression was mainly assessed on a plethora of equivalent neural-like cell lines *in vitro*: primary cortical neurons [55, 102], SH-SY5Y and N2a neuroblastoma cells [51, 55, 103–106], HN33 mouse hippocampal neuronal cells x neuroblastoma cells [107, 108], PC12 pheochromocytoma cells [52], human H4 neuroglioma cells [109], human U87 and U251 glioblastoma cells [110], and mouse HT22 hippocampal neuronal cells [58] (Figure 2). Moreover, as Ngb has been linked to cytoprotection and the hypoxia response in general, nonneural-like cell lines were successfully transfected to also overexpress Ngb, including rat H9c2 cardiomyocytes [111], HepG2 human liver cancer cells [112], and the well-characterised human embryonic kidney HEK293 cells [113].

In a study to determine the role of Ngb in oxygen and glucose deprivation (OGD), Yu et al. created murine Ngb-overexpressing primary mouse cortical neurons using the adenoassociated vector pACP. Adenoassociated vectors are widely used (described in Section 3.2) as they are highly effective in transducing dividing and nondividing cells. They provide stable gene expression, although they usually do not integrate into the genome [114]. Transduced mouse primary cortical neurons showed a 4.6-fold increase in Ngb, resulting in significantly reduced OGD-induced neuron death that could, at least in part, be ascribed to mitochondrial mechanisms ([102], Figure 4). Another viral vector-based transduction was carried out by Zhang et al. using the retroviral pMMP vector to overexpress human Ngb in human U87 glioblastoma cells. An increased cell proliferation and apoptosis resistance was reported, which could be attributed to about 2.7-fold increased Ngb expression levels ([110], Figure 4). Retroviral vectors are also widely used because they have the same advantages as the adenoassociated vectors plus a higher transduction efficiency. However, they randomly integrate into the host genome, which may lead to interruption of essential host genes [114]. Interpretation of data could become ambiguous as it is difficult to determine whether the resulting biological phenotype is caused by Ngb overexpression or by gene interruption in the host.

Besides the use of viral vector technology, high levels of stable and transient Ngb overexpression can be obtained in mammalian host cells using CMV promoter-driven overexpression vectors such as pEGFP-N1 and pcDNA3.1 [52, 55, 103–105, 107–109, 111–113]. Transient transfection can be used to generate and investigate the relatively short-term impact of Ngb overexpression. Li et al. created an Alzheimer's disease model where PC12 cells were pretreated with A*β*. This cell line was transiently transfected using pcDNA3.1-hNgb, and a decrease in the levels of A*β*-induced ROS and lipid peroxidation was reported, supporting the effectiveness of transient transfections ([115], Figure 4). They showed that the higher the pcDNA3.1-hNgb concentrations used (0.5, 1.0, and 2.0 *μ*g per 10^6^ cells), the higher the Ngb mRNA and protein expression levels (±4-fold, ±8-fold, and ±16-fold, respectively, compared to the empty vector). However, repeated transfections might be needed to maintain the level of Ngb overexpression [52]. To assure stable Ngb overexpression levels, providing long-term expression of the exogenous genetic material, the transfected genetic material is integrated into the host cell genome under antibiotic selection.

An added value of the use of the pEGFP-N1 vector is the presence of (E)GFP to evaluate the transfection efficiency. In line with the latter, the pcDEST40 expression vector assesses high overexpression levels of Ngb in a transient way, tagged by a His-tag and V5 epitope-tag [51, 106]. Additionally, validated antibodies are available for reporter molecules such as EGFP, His-tag, and V5-tag, providing an extra control for ambiguous Ngb specific antibodies. Indeed, a critical point already known in the field encompasses a low antigenicity of Ngb, resulting in antibodies to be generally generated in low titres and with aspecific cross-reactivity (e.g., against triose-phosphate isomerase) [116]. As such, when validated on brain sections of Ngb-null mice, some antibodies were even shown to display a widespread staining pattern [117]. Mention has also been made of noncorrelating values between Ngb transcript and protein levels [118]. Together, these results should prompt us to better characterise Ngb-specific antibodies and probes or to use alternative detection methods. As discussed previously in the *in vivo* model section of this review, an alternate adenoassociated expression vector exists that provides stable overexpression of Ngb under the strong chicken *β*-actin promoter and CMV enhancer: the pTR-UF12d expression vector. Incorporation of a GFP sequence provides an extra validation of the overexpression of Ngb and GFP and can analogously be used for the determination of transfection efficiency [58].

The majority of studies report the Ngb overexpression level in comparison to empty vector transfected cells or relative to endogenous mRNA and/or protein Ngb levels in a specific cell type [52, 58, 103–108, 111, 112]. Antao et al. explored the effect of Ngb overexpression in SH-SY5Y cells after H_2_O_2_ insult and reported these results in a different manner. They referred to the *in vivo* situation in the brain by calculating the Ngb concentration in the SH-SY5Y model and comparing it to brain Ngb concentrations. In this manner, Antao et al. reported neuroprotective effects of Ngb after H_2_O_2_ insult by reducing oxidative stress and increasing intracellular ATP concentrations. The concentration of the Ngb fusion protein was 13 mg/200 ml cell lysate corresponding to 3.7 *μ*M, which is approximately 4-fold greater than the estimated level in the brain [29, 51].

### 4.3. Alternative Ways of Ngb Delivery

As already described in Section 3.2, CPPs have been considered to be of great value for delivering proteins across cell membranes and across the blood-brain barrier [83]. Two CPPs have been reported in the Ngb research field, TAT [83] and Chariot/Pep-1 [119]. They have been used to transduce neural-like cells such as PC12 pheochromocytoma cells [120], retinal ganglion cell line RGC-5 cells [121], SH-SY5Y neuroblastoma cells [121], and primary rat cortical neurons [122], as well as nonneural-like cells such as human pancreatic islets [123]. In contrast to gene delivery by viral or nonviral vectors, the intracellular Ngb delivery is not expressed as increased fold change of mRNA or protein expression level. It is rather reported as a specific concentration of CPP-Ngb administered to the cells, generally about 0.2–2 *μ*M CPP-Ngb. In addition, most of the studies evaluate the uptake of the construct by screening for the FITC label attached to CPP-Ngb by immunocytochemistry, flow cytometry, or western blot [120–123]. In general, studies using CPP-Ngb delivery are focussed on the role of Ngb in hypoxia and oxidative stress. An important note should be considered from the study by Zhou et al. in which 48 hours after TAT-Ngb delivery, no FITC signal was detected, suggesting a transient Ngb presence. The relatively fast decrease in Ngb levels should be taken into consideration during time-dependent hypoxic insults or oxidative stress on cells [122]. While Peroni et al. did not report neuroprotective effects of Ngb in RGC-5 and SH-SY5Y cells 12 hours or 18 hours under OGD [121], Zhou et al. and Mendoza et al. reported beneficial effects of Ngb after 24 hours of hypoxia and oxidative stress ([123], Figure 4). As different concentrations of CPP-Ngb and different periods of hypoxia and OGD were used to induce the Ngb neuroprotective effects, it is difficult to compare the different studies [120–123].

Another way to mimic and assess the effects of intracellular overexpression of Ngb in neurons is to incubate Ngb with purified mouse neuronal mitochondria ([102], Figure 4). Yu et al. reported inhibition of NAD^+^ release and Cyt c release due to excesses of Ngb [102]. However, this is an artificial setup, which should only really be used to explore some of the mechanistic details but not to form first conclusions. In addition, the recombinant chimeric ZHHH Ngb can be used *in vitro* as well as *in vivo*, making direct correlations achievable (Section 3.2) [80, 81, 124].

### 4.4. Vector Technology in Ngb Knockdown Experiments *In Vitro*

Analogous to *in vivo* Ngb silencing, the use of RNAi has been shown to be a powerful tool to investigate the functional significance of Ngb *in vitro* (Figure 2). In general, RNAi is based on the sequence-specific degradation of host mRNA after the recognition by double-stranded RNA that is identical to the target sequence, as described in Section 3.4 [125]. In the Ngb research field, short interfering RNA (siRNA) and shRNA are mainly used [48, 55, 102, 110–112, 115, 126, 127]. The simplest approach for RNAi is to directly transfect siRNA into the cytosol [46, 48, 115, 126]. However, this technique has limitations as not every cell type can be transfected as easily as another, leading to variations in transfection efficiency [98]. Nayak et al. transfected primary turtle neuronal cultures with EGFP constructs to evaluate their setup and to determine the probable transfection efficiency rate of their *anti-turtle-Ngb* siRNA [126]. This method is considered reliable as it is widely used in similar Ngb knockdown studies [55, 102, 126]. On the other hand, shRNA is widely used to silence Ngb expression [55, 102, 111, 112]. Several studies use the GFP-expressing p-Genesil-1 expression plasmid vector encoding shRNA of *Ngb* transcribed by the RNA polymerase III hU6 promoter [102, 111, 112]. Yu et al. inserted the shRNA against mouse *Ngb* in the retroviral plasmid pGFP-VRS to transduce primary mouse cortical neurons [102]. In general, the use of shRNA is favoured over siRNA. The latter requires high concentrations of direct delivery, leading to more off-site nonspecific effects. Furthermore, whereas shRNA provides a stable knockdown mechanism, siRNA is only stable for 48 hours, making repeated transfections necessary to overcome its transient nature. Also, selection by drug resistance can be time-consuming. Therefore, evaluation of Ngb expression on transcript and protein levels is necessary to determine the reliability of shRNA and siRNA models. In general, scrambled siRNA sequences or empty shRNA vectors (e.g., p-Genesil-1) are used as a control [48, 55, 102, 110–112, 115, 126, 127]. RNAi methodologies created Ngb-deficient cellular models that showed increased susceptibility for OGD, oxidative stress and apoptosis [48, 111, 115, 126, 127], suppression of neuronal development [55], reduction of retinal homeostasis [48], and ambiguous effects on cancer cells [110, 112].

Another way to create an *in vitro* cell line lacking Ngb is by using CRE-Lox recombination. Ngb has already been suggested to affect neuronal development, an observation which is supported by relatively low Ngb expression levels in the early stages of mouse brain development, which then increase during the later developmental stages and even increase further up to the young adult stage. Hence, it is of great value to investigate the functional significance of Ngb in this process using NSCs. Luyckx et al. generated a Ngb_fl_ mouse model targeting exons 2 and 3 of the *Ngb* locus by inserting *loxP* sites. Successful *in vitro* CRE-Lox recombination allowed the investigation of the characteristics of these Ngb knockout NSCs and unravelled CDKN1A/CDK6-dependent increased proliferation of NSCs due to the loss of Ngb [128].

In contrast to unanimous results of Ngb in overexpression models, supporting the neuroprotective hypothesis, *in vivo* studies on Ngb knockout models do not support this hypothesis for endogenously expressed Ngb ([67–69, 73], Figure 4). In addition, in contrast to these *in vivo* Ngb knockout studies, *in vitro* studies on Ngb knockdown cells do claim a role of endogenous Ngb in protection against oxidative stress, oxygen deprivation [102, 115, 126], and apoptosis [111], supporting retinal homeostasis [48] and neuronal development ([55], Figure 4). Concerning the latter, Luyckx et al. reported increased growth proliferation in NSCs of which the role in neuronal development is still unknown [128]. Intriguingly, ambiguous results were found in cancer research. Although Zhang et al. reported that Ngb knockdown promoted human HCC cell line growth and proliferation and tumour growth *in vivo* through the RAF/MEK/ERK pathway, another research group of Zhang and colleagues stated that Ngb knockdown retained U251 glioma cell growth and facilitated apoptosis ([110, 112], Figure 4). However, the study of Zhang et al. in 2013 showed downregulation of endogenous Ngb in hepatocellular carcinoma while Ngb is reported to be upregulated in glioma in the study of Zhang et al. in 2017 [110, 112]. These observations support the plethora of functions which Ngb may exert. On the one hand, Ngb can support hypoxia-mediated defences to allow cancer cells to adapt to the tumour microenvironment, and on the other, it may enable tumour suppressor capacities in other malignant cells [33, 110, 112, 129, 130].

## 5. Other Regulators of Ngb Gene Expression

Ngb's overexpression or ectopic expression elicits survival-promoting cytoprotective effects in different pathologies in nervous and nonnervous tissues. Thus, injured tissues might benefit from therapeutic administration or induction of Ngb expression (Table 1). Targeted intracellular Ngb delivery or upregulation would be of great value as Ngb, except for zebrafish Ngb, is membrane impermeable [80, 124].

Hemin, the ferric chloride salt of haem, which is used for the treatment of porphyria attacks, has already been described to stimulate expression of Hb and Mb [131, 132]. In addition, Zhu et al. demonstrated that Ngb is a hemin-inducible gene in neural cells through the sGC-PKG pathway [133]. Furthermore, deferoxamine, a cobalt and iron chelator that is used to treat iron poisoning, appeared capable of inducing Ngb protein expression in cultured neurons [107]. In addition, Jin et al. described that HN33 cells showed increased Ngb protein levels when they were cultured in the presence of the short-chain fatty acids cinnamic acid and valproic acid [134]. Cinnamic acid derivatives have been reported to have antioxidant and antimicrobial properties, making them promising therapeutic compounds [135]. Valproic acid is an anticonvulsant drug used in the treatment of patients with seizure disorders [136]. Zara et al. showed promising results with the administration of an ibuprofen and lipoic acid conjugate to rats suffering from Alzheimer's disease, promoting the maintenance of Ngb levels that were similar to the control group, enabling Ngb to perform neuroprotective and survival-promoting actions [53]. Furthermore, Ngb should also be considered to be a hormone-inducible protein that promotes cytoprotection after upregulation. It has been linked to several hormones such as a glycoproteic hormone erythropoietin [137, 138], thyroid hormones (THs) [118], and 17*β*-estradiol (E2), an oestrogen steroid hormone. Recently, the modulation of Ngb expression levels by the latter has gained a lot of interest, as elevation of Ngb levels by E2 has been reported in several *in vitro* models such as in human neuroblastoma cell lines [26, 39, 42], in mouse primary hippocampal neurons [139], and in astrocytes [140, 141]. The functional significance of this E2-induced upregulation of Ngb has been reported to play a role in the neuroprotective effect reflected by, for example, the protection against H_2_O_2_-induced apoptosis [42, 139, 140] and the anti-inflammatory effect in astrocytes [141]. Moreover, recently, Ngb relocalisation to the mitochondria was reported after the effect of E2 stimulation and H_2_O_2_ exposure enabling Ngb to interact with cytochrome c in the mitochondria, preventing release into the cytosol [32, 39].

Nevertheless, compounds that can pharmacologically or biologically raise Ngb levels must induce subtoxic Ngb levels to preserve cellular homeostasis and, in addition, still manage to achieve sufficient doses to promote cell protection and survival.

## 6. The Future of Ngb Expression Models

Eighteen years after the discovery of Ngb, it is clear that many questions remain unanswered about the biological significance of this protein. Numerous technologies have been used to achieve a plethora of Ngb *in vivo* and *in vitro* expression systems (Figure 2). Although concrete modes of action and major functionalities of Ngb remain elusive, a toolbox of models has been composed. As the level of endogenous Ngb expression and overexpression differs between models, interpretation of a comparative study is ambitious and limited, though not always essential. Moreover, Ngb-inducible insults such as hypoxia, ischemia, and oxidative stress *in vivo* markedly differ from the *in vitro* setups. Furthermore, these models highly differ from specific neurological injury and neurodegenerative disease models. Different secondary survival-promoting mechanisms may be affected, depending on the composition of the microenvironment of the tissues or cells. Furthermore, especially for inborn Ngb knockout animal models, compensatory mechanisms may have been induced during development, masking the effect of Ngb deficiency [86].

Remarkably, Ngb overexpression model systems all point to a role for Ngb in neuroprotection and cytoprotection in general. However, Ngb-deficient models show ambiguous results and hence do not indicate a specific endogenous function for Ngb. In the light of these variations, it is thought that Ngb has widespread biological activities that need to be evaluated thoroughly with specific validated controls relevant to the specific experimental conditions.

As Ngb is a multifunctional protein, which affects various signalling pathways, Ngb research will remain challenging. In that view, the present toolbox of diverse expression models will be of great value to the Ngb research community.

## Figures and Tables

**Table 1:** Positive *in vitro* and/or *in vivo* modulators of human and rodent Ngb levels.

Modulators	mRNA	Protein	References
Hemin	+^∗^	+^∗^	[133]
Deferoxamine	+^∗^	+^∗^	[107, 134]
Short-chain fatty acids	+^∗^	+^∗^	[134]
Ibuprofen and R-*α*-lipoic acid conjugate	ND	+^#^	[53]
17*β*-Estradiol	+^∗^	+^∗^	[26, 32, 139, 141, 142]
EPO	+^#^	+^#^	[138]
TH	+^#^	+^#^	[118]

EPO: erythropoietin; TH: thyroid hormone; ND: not determined. ^∗^ and ^#^ indicate *in vitro* and *in vivo* studies, respectively. Adapted from Ascenzi et al. [129]..

## Abbreviations

A*β*: Amyloid beta
APP: Amyloid precursor protein
CMV: Cytomegalovirus
CPP: Cell-penetrating peptide
Cyt c: Cytochrome c
Cyt c_1_: Cytochrome c_1_
EPO: Erythropoietin
ETC: Electron transport chain
E2: 17*β*-Estradiol
FITC: Fluorescein isothiocyanate
GDI: Guanosine nucleotide dissociation inhibitor
GDP: Guanosine diphosphate
(E)GFP: Enhanced green fluorescent protein
Hb: Haemoglobin
HisE7: Histidine at position 7 of the E-helix
HTT: Huntingtin
LADCAO: Left anterior descending coronary artery occlusion
Mb: Myoglobin
MCAO: Middle cerebral artery occlusion
mPTP: Mitochondrial permeability transition pores
ND: Not determined
Ngb: Neuroglobin
m, h, r, rbNgb: Mouse, human, rat, rabbit neuroglobin
Ngb_fl_: Neuroglobin-floxed
NMDA: Methyl-D-aspartate
NSC: Neural stem cell
O_2_: Oxygen
OGD: Oxygen and glucose deprivation
PI3K: Phosphatidylinositol 3-kinase
PD: Parkinson's disease
PS-ODN: Phosphorothioate antisense oligodeoxynucleotide
PS1: Presenilin-1
RAC-1: Ras-related C3 botulinum toxin substrate 1
shRNAs: Short hairpin RNA
siRNA: Small interfering RNA
RNAi: RNA interference
RNS: Reactive nitrogen species
ROS: Reactive oxygen species
TAT: 11-Amino-acid human immunodeficiency virus transactivator of transcription
TBI: Traumatic brain injury
TH: Thyroid hormone
VDAC: Voltage-dependent anion channel
WT: Wild type.

## References

[1] Acker T., Acker H. (2004). Cellular oxygen sensing need in CNS function: physiological and pathological implications. *Journal of Experimental Biology*.

[2] Burmester T., Hankeln T. (2014). Function and evolution of vertebrate globins. *Acta Physiologica*.

[3] Vinogradov S. N., Moens L. (2008). Diversity of globin function: enzymatic, transport, storage, and sensing. *Journal of Biological Chemistry*.

[4] Burmester T., Weich B., Reinhardt S., Hankeln T. (2000). A vertebrate globin expressed in the brain. *Nature*.

[5] Wittenberg B. A., Briehl R. W., Wittenberg J. B. (1965). Haemoglobins of invertebrate tissues. Nerve haemoglobins of Aphrodite, Aplysia and Halosydna. *Biochemical Journal*.

[6] Lankester E. R. (1872). I. A contribution to the knowledge of haemoglobin. *Proceedings of the Royal Society of London*.

[7] Geuens E., Dewilde S., Hoogewijs D. (2004). Nerve globins in invertebrates. *IUBMB Life*.

[8] Burmester T., Ebner B., Weich B., Hankeln T. (2002). Cytoglobin: a novel globin type ubiquitously expressed in vertebrate tissues. *Molecular Biology and Evolution*.

[9] Richter F., Meurers B. H., Zhu C., Medvedeva V. P., Chesselet M.-F. (2009). Neurons express hemoglobin *α*‐ and *β*‐chains in rat and human brains. *The Journal of Comparative Neurology*.

[10] Schelshorn D. W., Schneider A., Kuschinsky W. (2008). Expression of hemoglobin in rodent neurons. *Journal of Cerebral Blood Flow & Metabolism*.

[11] Avivi A., Gerlach F., Joel A. (2010). Neuroglobin, cytoglobin, and myoglobin contribute to hypoxia adaptation of the subterranean mole rat *Spalax*. *Proceedings of the National Academy of Sciences of the United States of America*.

[12] Puspitaningrum R., Wanandi S. I., Soegianto R. R., Sadikin M., Williams D. R., Cossins A. R. (2010). Myoglobin expression in Chelonia mydas brain, heart and liver tissues. *HAYATI Journal of Biosciences*.

[13] Pesce A., De Sanctis D., Nardini M. (2004). Reversible hexa- to penta-coordination of the heme Fe atom modulates ligand binding properties of neuroglobin and cytoglobin. *IUBMB Life*.

[14] Vallone B., Nienhaus K., Brunori M., Nienhaus G. U. (2004). The structure of murine neuroglobin: novel pathways for ligand migration and binding. *Proteins*.

[15] Dewilde S., Kiger L., Burmester T. (2001). Biochemical characterization and ligand binding properties of neuroglobin, a novel member of the globin family. *Journal of Biological Chemistry*.

[16] Pesce A., Bolognesi M., Bocedi A. (2002). Neuroglobin and cytoglobin: fresh blood for the vertebrate globin family. *EMBO Reports*.

[17] Hankeln T., Ebner B., Fuchs C. (2005). Neuroglobin and cytoglobin in search of their role in the vertebrate globin family. *Journal of Inorganic Biochemistry*.

[18] Fago A., Hundahl C., Dewilde S., Gilany K., Moens L., Weber R. E. (2004). Allosteric regulation and temperature dependence of oxygen binding in human neuroglobin and cytoglobin: molecular mechanisms and physiological significance. *Journal of Biological Chemistry*.

[19] Herold S., Fago A., Weber R. E., Dewilde S., Moens L. (2004). Reactivity studies of the Fe(III) and Fe(II)NO forms of human neuroglobin reveal a potential role against oxidative stress. *Journal of Biological Chemistry*.

[20] Wystub S., Ebner B., Fuchs C., Weich B., Burmester T., Hankeln T. (2004). Interspecies comparison of neuroglobin, cytoglobin and myoglobin: sequence evolution and candidate regulatory elements. *Cytogenetic and Genome Research*.

[21] Cutrupi S., Ferrero G., Reineri S., Cordero F., De Bortoli M. (2014). Genomic lens on neuroglobin transcription. *IUBMB Life*.

[22] Fabrizius A., Andre D., Laufs T. (2016). Critical re-evaluation of neuroglobin expression reveals conserved patterns among mammals. *Neuroscience*.

[23] Reuss S., Saaler-Reinhardt S., Weich B. (2002). Expression analysis of neuroglobin mRNA in rodent tissues. *Neuroscience*.

[24] Schmidt M., Giessl A., Laufs T., Hankeln T., Wolfrum U., Burmester T. (2003). How does the eye breathe?: evidence for neuroglobin-mediated oxygen supply in the mammalian retina. *Journal of Biological Chemistry*.

[25] Fiocchetti M., Cipolletti M., Leone S. (2016). Neuroglobin in breast cancer cells: effect of hypoxia and oxidative stress on protein level, localization, and anti-apoptotic function. *PLoS One*.

[26] Guglielmotto M., Reineri S., Iannello A. (2016). E2 regulates epigenetic signature on neuroglobin enhancer-promoter in neuronal cells. *Frontiers in Cellular Neuroscience*.

[27] Zhang C., Wang C., Deng M. (2002). Full-length cDNA cloning of human neuroglobin and tissue expression of rat neuroglobin. *Biochemical and Biophysical Research Communications*.

[28] Geuens E., Brouns I., Flamez D., Dewilde S., Timmermans J.-P., Moens L. (2003). A globin in the nucleus!. *Journal of Biological Chemistry*.

[29] Wystub S., Laufs T., Schmidt M. (2003). Localization of neuroglobin protein in the mouse brain. *Neuroscience Letters*.

[30] Hankeln T., Wystub S., Laufs T. (2004). The Cellular and Subcellular Localization of Neuroglobin and Cytoglobin ‐ a Clue to Their Function?. *IUBMB Life*.

[31] Hundahl C. A., Allen G. C., Hannibal J. (2010). Anatomical characterization of cytoglobin and neuroglobin mRNA and protein expression in the mouse brain. *Brain Research*.

[32] De Marinis E., Fiocchetti M., Acconcia F., Ascenzi P., Marino M. (2013). Neuroglobin upregulation induced by 17*β*-estradiol sequesters cytocrome c in the mitochondria preventing H_2_O_2_-induced apoptosis of neuroblastoma cells. *Cell Death & Disease*.

[33] Burmester T., Hankeln T. (2009). What is the function of neuroglobin?. *Journal of Experimental Biology*.

[34] Van Acker Z. P., Luyckx E., Dewilde S. (2019). Neuroglobin expression in the brain: a story of tissue homeostasis preservation. *Molecular Neurobiology*.

[35] Brunori M., Giuffrè A., Nienhaus K., Nienhaus G. U., Scandurra F. M., Vallone B. (2005). Neuroglobin, nitric oxide, and oxygen: functional pathways and conformational changes. *Proceedings of the National Academy of Sciences of the United States of America*.

[36] Yu Z., Xu J., Liu N. (2012). Mitochondrial distribution of neuroglobin and its response to oxygen–glucose deprivation in primary-cultured mouse cortical neurons. *Neuroscience*.

[37] Yu Z., Liu N., Wang Y., Li X., Wang X. (2012). Identification of neuroglobin-interacting proteins using yeast two-hybrid screening. *Neuroscience*.

[38] Fago A., Mathews A. J., Moens L., Dewilde S., Brittain T. (2006). The reaction of neuroglobin with potential redox protein partners cytochrome b-5 and cytochrome c. *FEBS Letters*.

[39] Fiocchetti M., Nuzzo M. T., Totta P., Acconcia F., Ascenzi P., Marino M. (2014). Neuroglobin, a pro-survival player in estrogen receptor *α*-positive cancer cells. *Cell Death & Disease*.

[40] Fiocchetti M., Camilli G., Acconcia F., Leone S., Ascenzi P., Marino M. (2015). ER*β*-dependent neuroglobin up-regulation impairs 17*β*-estradiol-induced apoptosis in DLD-1 colon cancer cells upon oxidative stress injury. *The Journal of Steroid Biochemistry and Molecular Biology*.

[41] Nuzzo M. T., Marino M. (2016). Estrogen/huntingtin: a novel pathway involved in neuroprotection. *Neural Regeneration Research*.

[42] Nuzzo M. T., Fiocchetti M., Totta P. (2017). Huntingtin polyQ mutation impairs the 17*β*-estradiol/neuroglobin pathway devoted to neuron survival. *Molecular Neurobiology*.

[43] Fiocchetti M., Cipolletti M., Brandi V., Polticelli F., Ascenzi P. (2017). Neuroglobin and friends. *Journal of Molecular Recognition*.

[44] Wakasugi K., Nakano T., Morishima I. (2003). Oxidized human neuroglobin acts as a heterotrimeric G*α* protein guanine nucleotide dissociation inhibitor. *Journal of Biological Chemistry*.

[45] Watanabe S., Takahashi N., Uchida H., Wakasugi K. (2012). Human neuroglobin functions as an oxidative stress-responsive sensor for neuroprotection. *The Journal of Biological Chemistry*.

[46] Khan A. A., Mao X. O., Banwait S., Jin K., Greenberg D. A. (2007). Neuroglobin attenuates *β*-amyloid neurotoxicity *in vitro* and transgenic Alzheimer phenotype *in vivo*. *Proceedings of the National Academy of Sciences of the United States of America*.

[47] Khan A. A., Mao X. O., Banwait S. (2008). Regulation of hypoxic neuronal death signaling by neuroglobin. *The FASEB journal*.

[48] Lechauve C., Augustin S., Cwerman-Thibault H. (2012). Neuroglobin involvement in respiratory chain function and retinal ganglion cell integrity. *Biochimica et Biophysica Acta (BBA) - Molecular Cell Research*.

[49] Bentmann A., Schmidt M., Reuss S., Wolfrum U., Hankeln T., Burmester T. (2005). Divergent distribution in vascular and avascular mammalian retinae links neuroglobin to cellular respiration. *Journal of Biological Chemistry*.

[50] Garofalo T., Ferri A., Sorice M. (2018). Neuroglobin overexpression plays a pivotal role in neuroprotection through mitochondrial raft-like microdomains in neuroblastoma SK-N-BE2 cells. *Molecular and Cellular Neuroscience*.

[51] Antao S. T., Duong T. T. H., Aran R., Witting P. K. (2010). Neuroglobin overexpression in cultured human neuronal cells protects against hydrogen peroxide insult *via* activating phosphoinositide-3 kinase and opening the mitochondrial K_ATP_ channel. *Antioxidants & Redox Signaling*.

[52] Li R. C., Pouranfar F., Lee S. K., Morris M. W., Wang Y., Gozal D. (2008). Neuroglobin protects PC12 cells against *β*-amyloid-induced cell injury. *Neurobiology of Aging*.

[53] Zara S., De Colli M., Rapino M. (2013). Ibuprofen and lipoic acid conjugate neuroprotective activity is mediated by Ngb/Akt intracellular signaling pathway in Alzheimer’s disease rat model. *Gerontology*.

[54] Li Y., Dai Y.-b., Sun J.-y. (2016). Neuroglobin attenuates beta amyloid-induced apoptosis through inhibiting caspases activity by activating PI3K/Akt signaling pathway. *Journal of Molecular Neuroscience*.

[55] Li L., Liu Q. R., Xiong X. X. (2014). Neuroglobin promotes neurite outgrowth via differential binding to PTEN and Akt. *Molecular Neurobiology*.

[56] Wen H., Liu L., Zhan L. (2018). Neuroglobin mediates neuroprotection of hypoxic postconditioning against transient global cerebral ischemia in rats through preserving the activity of Na^+^/K^+^ ATPases. *Cell Death & Disease*.

[57] Wakasugi K., Kitatsuji C., Morishima I. (2005). Possible neuroprotective mechanism of human neuroglobin. *Annals of the New York Academy of Sciences*.

[58] Cai B., Li W., Mao X. (2016). Neuroglobin overexpression inhibits AMPK signaling and promotes cell anabolism. *Molecular Neurobiology*.

[59] Khan A. A., Wang Y., Sun Y. (2006). Neuroglobin-overexpressing transgenic mice are resistant to cerebral and myocardial ischemia. *Proceedings of the National Academy of Sciences of the United States of America*.

[60] Khan A. A., Sun Y., Jin K. (2007). A neuroglobin-overexpressing transgenic mouse. *Gene*.

[61] Chan A. S. Y., Saraswathy S., Rehak M., Ueki M., Rao N. A. (2012). Neuroglobin protection in retinal ischemia. *Investigative Ophthalmology & Visual Science*.

[62] Raida Z., Hundahl C. A., Nyengaard J. R., Hay-Schmidt A. (2013). Neuroglobin over expressing mice: expression pattern and effect on brain ischemic infarct size. *PLoS One*.

[63] Van Leuven W., Van Dam D., Moens L., De Deyn P. P., Dewilde S. (2013). A behavioural study of neuroglobin-overexpressing mice under normoxic and hypoxic conditions. *Biochimica et Biophysica Acta (BBA) - Proteins and Proteomics*.

[64] Luyckx E., Everaert B. R., Van der Veken B. (2018). Cytoprotective effects of transgenic neuroglobin overexpression in an acute and chronic mouse model of ischemic heart disease. *Heart and Vessels*.

[65] Davis J., Maillet M., Miano J. M., Molkentin J. D. (2012). Lost in transgenesis: a users guide for genetically manipulating the mouse in cardiac research. *Circulation research*.

[66] Williams R. S., Wagner P. D. (2000). Transgenic animals in integrative biology: approaches and interpretations of outcome. *Journal of Applied Physiology*.

[67] Wang X., Liu J., Zhu H. (2008). Effects of neuroglobin overexpression on acute brain injury and long-term outcomes after focal cerebral ischemia. *Stroke*.

[68] Zhao S., Yu Z., Zhao G. (2012). Neuroglobin-overexpression reduces traumatic brain lesion size in mice. *BMC Neuroscience*.

[69] Taylor J. M., Kelley B., Gregory E. J., Berman N. E. J. (2014). Neuroglobin overexpression improves sensorimotor outcomes in a mouse model of traumatic brain injury. *Neuroscience Letters*.

[70] Li R. C., Guo S. Z., Lee S. K., Gozal D. (2010). Neuroglobin protects neurons against oxidative stress in global ischemia. *Journal of Cerebral Blood Flow & Metabolism*.

[71] Lee H. M., Greeley G. H., Englander E. W. (2011). Transgenic overexpression of neuroglobin attenuates formation of smoke-inhalation-induced oxidative DNA damage, in vivo, in the mouse brain. *Free Radical Biology and Medicine*.

[72] Gorgun F. M., Zhuo M., Singh S., Englander E. W. (2014). Neuroglobin mitigates mitochondrial impairments induced by acute inhalation of combustion smoke in the mouse brain. *Inhalation toxicology*.

[73] Shang A., Feng X., Wang H. (2012). Neuroglobin upregulation offers neuroprotection in traumatic brain injury. *Neurological Research*.

[74] Nayerossadat N., Maedeh T., Ali P. A. (2012). Viral and nonviral delivery systems for gene delivery. *Advanced Biomedical Research*.

[75] Sun Y., Jin K., Peel A., Mao X. O., Xie L., Greenberg D. A. (2003). Neuroglobin protects the brain from experimental stroke in vivo. *Proceedings of the National Academy of Sciences of the United States of America*.

[76] Lechauve C., Augustin S., Cwerman-Thibault H. (2014). Neuroglobin gene therapy prevents optic atrophy and preserves durably visual function in Harlequin mice. *Molecular Therapy*.

[77] Cwerman-Thibault H., Lechauve C., Augustin S. (2017). Neuroglobin can prevent or reverse glaucomatous progression in DBA/2J mice. *Molecular Therapy Methods & Clinical Development*.

[78] Tao Y., Yang Z., Fang W., Ma Z., Huang Y. F., Li Z. (2017). Adeno-associated virus-mediated neuroglobin overexpression ameliorates the N-methyl-N-nitrosourea-induced retinal impairments: a novel therapeutic strategy against photoreceptor degeneration. *Therapeutics and Clinical Risk Management*.

[79] Chen X. W., Lin W. P., Lin J. H. (2011). The protective effects of the lentivirus-mediated neuroglobin gene transfer on spinal cord injury in rabbits. *Spinal Cord*.

[80] Watanabe S., Wakasugi K. (2008). Zebrafish neuroglobin is a cell-membrane-penetrating globin. *Biochemistry*.

[81] Kamioka Y., Fujikawa C., Ogai K. (2013). Functional characterization of fish neuroglobin: zebrafish neuroglobin is highly expressed in amacrine cells after optic nerve injury and can translocate into ZF4 cells. *Biochimica et Biophysica Acta (BBA) - Proteins and Proteomics*.

[82] Sugitani K., Koriyama Y., Sera M., Arai K., Ogai K., Wakasugi K. (2017). A novel function of neuroglobin for neuroregeneration in mice after optic nerve injury. *Biochemical and Biophysical Research Communications*.

[83] Schwarze S. R., Ho A., Vocero-Akbani A., Dowdy S. F. (1999). In vivo protein transduction: delivery of a biologically active protein into the mouse. *Science*.

[84] Dietz G. P. H. (2011). Protection by neuroglobin and cell-penetrating peptide-mediated delivery in vivo: a decade of research. *Experimental Neurology*.

[85] Cai B., Lin Y., Xue X.-H., Fang L., Wang N., Wu Z.-Y. (2011). TAT-mediated delivery of neuroglobin protects against focal cerebral ischemia in mice. *Experimental Neurology*.

[86] Hundahl C. A., Luuk H., Ilmjärv S. (2011). Neuroglobin-deficiency exacerbates Hif1A and c-FOS response, but does not affect neuronal survival during severe hypoxia in vivo. *PLoS One*.

[87] Raida Z., Hundahl C. A., Kelsen J., Nyengaard J. R., Hay-Schmidt A. (2012). Reduced infarct size in neuroglobin-null mice after experimental stroke in vivo. *Experimental & Translational Stroke Medicine*.

[88] Ilmjärv S., Reimets R., Hundahl C. A., Luuk H. (2014). Effect of light on global gene expression in the neuroglobin-deficient mouse retina. *Biomedical Reports*.

[89] Hundahl C. A., Fahrenkrug J., Hay-Schmidt A., Georg B., Faltoft B., Hannibal J. (2012). Circadian behaviour in neuroglobin deficient mice. *PLoS One*.

[90] Hundahl C. A., Fahrenkrug J., Luuk H., Hay-Schmidt A., Hannibal J. (2012). Restricted expression of neuroglobin in the mouse retina and co-localization with melanopsin and tyrosine hydroxylase. *Biochemical and Biophysical Research Communications*.

[91] Reuss S., Banica O., Elgurt M. (2016). Neuroglobin expression in the mammalian auditory system. *Molecular Neurobiology*.

[92] Vorasubin N., Hosokawa S., Hosokawa K., Ishiyama G., Ishiyama A., Lopez I. A. (2016). Neuroglobin immunoreactivity in the human cochlea. *Brain research*.

[93] Nowotny M., Kiefer L., Andre D., Fabrizius A., Hankeln T., Reuss S. (2017). Hearing without neuroglobin. *Neuroscience*.

[94] Skarnes W. C., Rosen B., West A. P. (2011). A conditional knockout resource for the genome-wide study of mouse gene function. *Nature*.

[95] Dias N., Stein C. A. (2002). Antisense oligonucleotides: basic concepts and mechanisms. *Molecular Cancer Therapeutics*.

[96] Fiset P.-O. G., Soussi A. (2001). Antisense oligonucleotides: problems with use and solutions. *Reviews in Biology and Biotechnology*.

[97] Watts J. K., Corey D. R. (2012). Silencing disease genes in the laboratory and the clinic. *Journal of Pathology*.

[98] Taxman D. J., Moore C. B., Guthrie E. H., Huang M. T.-H., Sioud M. (2010). Short hairpin RNA (shRNA): design, delivery, and assessment of gene knockdown. *RNA Therapeutics*.

[99] Vlachaki M. T., Hernandez-Garcia A., Ittmann M. (2002). Impact of preimmunization on adenoviral vector expression and toxicity in a subcutaneous mouse cancer model. *Molecular Therapy*.

[100] Liu J., Yu Z., Guo S. (2009). Effects of neuroglobin overexpression on mitochondrial function and oxidative stress following hypoxia/reoxygenation in cultured neurons. *Journal of Neuroscience Research*.

[101] Yu Z., Liu J., Guo S. (2009). Neuroglobin-overexpression alters hypoxic response gene expression in primary neuron culture following oxygen glucose deprivation. *Neuroscience*.

[102] Yu Z., Liu N., Li Y., Xu J., Wang X. (2013). Neuroglobin overexpression inhibits oxygen–glucose deprivation-induced mitochondrial permeability transition pore opening in primary cultured mouse cortical neurons. *Neurobiology of Disease*.

[103] Fordel E., Thijs L., Martinet W. (2006). Neuroglobin and cytoglobin overexpression protects human SH-SY5Y neuroblastoma cells against oxidative stress-induced cell death. *Neuroscience Letters*.

[104] Fordel E., Thijs L., Martinet W., Schrijvers D., Moens L., Dewilde S. (2007). Anoxia or oxygen and glucose deprivation in SH-SY5Y cells: a step closer to the unraveling of neuroglobin and cytoglobin functions. *Gene*.

[105] Raychaudhuri S., Skommer J., Henty K., Birch N., Brittain T. (2010). Neuroglobin protects nerve cells from apoptosis by inhibiting the intrinsic pathway of cell death. *Apoptosis*.

[106] Duong T. T. H., Witting P. K., Antao S. T. (2009). Multiple protective activities of neuroglobin in cultured neuronal cells exposed to hypoxia re-oxygenation injury. *Journal of Neurochemistry*.

[107] Sun Y., Jin K., Mao X. O., Zhu Y., Greenberg D. A. (2001). Neuroglobin is up-regulated by and protects neurons from hypoxic-ischemic injury. *Proceedings of the National Academy of Sciences of the United States of America*.

[108] Jin K., Mao X. O., Xie L., Khan A. A., Greenberg D. A. (2008). Neuroglobin protects against nitric oxide toxicity. *Neuroscience letters*.

[109] Kleinknecht A., Popova B., Lázaro D. F. (2016). C-terminal tyrosine residue modifications modulate the protective phosphorylation of serine 129 of *α*-synuclein in a yeast model of Parkinson’s disease. *PLoS Genetics*.

[110] Zhang B., Chang M., Wang J., Liu Y. (2017). Neuroglobin functions as a prognostic marker and promotes the tumor growth of glioma via suppressing apoptosis. *Biomedicine & Pharmacotherapy*.

[111] Liu Z.-F., Zhang X., Qiao Y.-X. (2015). Neuroglobin protects cardiomyocytes against apoptosis and cardiac hypertrophy induced by isoproterenol in rats. *International Journal of Clinical and Experimental Medicine*.

[112] Zhang J., Lan S. J., Liu Q. R., Liu J. M., Chen X. Q. (2013). Neuroglobin, a novel intracellular hexa-coordinated globin, functions as a tumor suppressor in hepatocellular carcinoma via Raf/MAPK/Erk. *Molecular Pharmacology*.

[113] Chen L. M., Xiong Y. S., Kong F. L. (2012). Neuroglobin attenuates Alzheimer-like tau hyperphosphorylation by activating Akt signaling. *Journal of Neurochemistry*.

[114] Kamimura K., Suda T., Zhang G., Liu D. (2011). Advances in gene delivery systems. *Pharmaceutical Medicine*.

[115] Li R. C., Morris M. W., Lee S. K., Pouranfar F., Wang Y., Gozal D. (2008). Neuroglobin protects PC12 cells against oxidative stress. *Brain Research*.

[116] Brittain T. (2012). The anti-apoptotic role of neuroglobin. *Cells*.

[117] Hundahl C. A., Kelsen J., Hay-Schmidt A. (2013). Neuroglobin and cytoglobin expression in the human brain. *Brain Structure and Function*.

[118] Oliveira K. C., da Conceição R. R., Piedade G. C. (2015). Thyroid hormone modulates neuroglobin and cytoglobin in rat brain. *Metabolic Brain Disease*.

[119] Morris M. C., Depollier J., Mery J., Heitz F., Divita G. (2001). A peptide carrier for the delivery of biologically active proteins into mammalian cells. *Nature Biotechnology*.

[120] Watanabe S., Wakasugi K. (2008). Neuroprotective function of human neuroglobin is correlated with its guanine nucleotide dissociation inhibitor activity. *Biochemical and Biophysical Research Communications*.

[121] Peroni D., Negro A., Bähr M., Dietz G. P. H. (2007). Intracellular delivery of neuroglobin using HIV-1 TAT protein transduction domain fails to protect against oxygen and glucose deprivation. *Neuroscience Letters*.

[122] Zhou G. Y., Zhou S.-N., Lou Z.-Y., Zhu C.-S., Zheng X.-P., Hu X.-Q. (2008). Translocation and neuroprotective properties of transactivator-of-transcription protein-transduction domain–neuroglobin fusion protein in primary cultured cortical neurons. *Biotechnology and Applied Biochemistry*.

[123] Mendoza V., Klein D., Ichii H. (2005). Protection of islets in culture by delivery of oxygen binding neuroglobin via protein transduction. *Transplantation Proceedings*.

[124] Watanabe S., Wakasugi K. (2010). Identification of residues critical for the cell-membrane-penetrating activity of zebrafish neuroglobin. *FEBS Letters*.

[125] Fire A., Xu S., Montgomery M. K., Kostas S. A., Driver S. E., Mello C. C. (1998). Potent and specific genetic interference by double-stranded RNA in Caenorhabditis elegans. *Nature*.

[126] Nayak G., Prentice H. M., Milton S. L. (2009). Role of neuroglobin in regulating reactive oxygen species in the brain of the anoxia-tolerant turtle Trachemys scripta. *Journal of Neurochemistry*.

[127] Ye S.-q., Zhou X.-y., Lai X.-j., Zheng L., Chen X.-q. (2009). Silencing neuroglobin enhances neuronal vulnerability to oxidative injury by down-regulating 14-3-3*γ*. *Acta Pharmacologica Sinica*.

[128] Luyckx E., Van Leuven W., Andre D. (2018). Loss of neuroglobin expression alters CDKN1A/CDK6-expression resulting in increased proliferation of neural stem cells. *Stem Cells and Development*.

[129] Ascenzi P., di Masi A., Leboffe L. (2016). Neuroglobin: from structure to function in health and disease. *Molecular Aspects of Medicine*.

[130] Emara M., Turner A. R., Allalunis-Turner J. (2010). Hypoxic regulation of cytoglobin and neuroglobin expression in human normal and tumor tissues. *Cancer Cell International*.

[131] Graber S. G., Woodworth R. C. (1986). Myoglobin expression in L6 muscle cells. Role of differentiation and heme. *Journal of Biological Chemistry*.

[132] Rutherford T. R., Clegg J. B., Weatherall D. J. (1979). K562 human leukaemic cells synthesise embryonic haemoglobin in response to haemin. *Nature*.

[133] Zhu Y., Sun Y., Jin K., Greenberg D. A. (2002). Hemin induces neuroglobin expression in neural cells. *Blood*.

[134] Jin K., Mao X. O., Lin X., Varghese J., Greenberg D. A. (2011). Pharmacological induction of neuroglobin expression. *Pharmacology*.

[135] Sova M. (2012). Antioxidant and antimicrobial activities of cinnamic acid derivatives. *Mini-Reviews in Medicinal Chemistry*.

[136] Macdonald R. L. M., J M. (1986). Anticonvulsant drugs: mechanisms of action. *Advances in Neurology*.

[137] Milano M., Collomp R. (2005). Erythropoietin and neuroprotection: a therapeutic perspective. *Journal of Oncology Pharmacy Practice*.

[138] Zhu L., Huang L., Wen Q., Wang T., Qiao L., Jiang L. (2017). Recombinant human erythropoietin offers neuroprotection through inducing endogenous erythropoietin receptor and neuroglobin in a neonatal rat model of periventricular white matter damage. *Neuroscience Letters*.

[139] De Marinis E., Ascenzi P., Pellegrini M. (2010). 17*β*-Estradiol – a new modulator of neuroglobin levels in neurons: role in neuroprotection against H_2_O_2_-induced toxicity. *Neurosignals*.

[140] Toro-Urrego N., Garcia-Segura L. M., Echeverria V., Barreto G. E. (2016). Testosterone protects mitochondrial function and regulates neuroglobin expression in astrocytic cells exposed to glucose deprivation. *Frontiers in Aging Neuroscience*.

[141] De Marinis E., Acaz-Fonseca E., Arevalo M. A. (2013). 17*β*-Oestradiol anti-inflammatory effects in primary astrocytes require oestrogen receptor *β*-mediated neuroglobin upregulation. *Journal of Neuroendocrinology*.

[142] De Marinis E., Marino M., Ascenzi P. (2011). Neuroglobin, estrogens, and neuroprotection. *IUBMB Life*.

